# Retinal findings in familial hypercholesterolemia: An exploratory case-control study

**DOI:** 10.1016/j.athplu.2026.100571

**Published:** 2026-06-04

**Authors:** Gisle Langslet, Synne Kollstad, Øystein Kalsnes Jørstad, Maja Gran Erke, Kjetil Retterstøl, Kirsten B. Holven

**Affiliations:** a Lipid Clinic, Oslo University Hospital, Oslo, Norway; b Department of Nutrition, Institute of Basic Medical Sciences, University of Oslo, Norway; c Department of Ophthalmology, Oslo University Hospital, Oslo, Norway; d Faculty of Medicine, University of Oslo, Oslo, Norway; e Norwegian Directorate of Health, Oslo, Norway; f Norwegian National Advisory Unit on Familial Hypercholesterolemia, Oslo University Hospital, Norway

## Abstract

**Background and aims:**

Patients with familial hypercholesterolemia (FH) have elevated LDL-C levels from birth and increased risk of cardiovascular disease due to atherosclerosis, mostly affecting larger arteries. Less is known about effects of elevated LDL-cholesterol on smaller arteries. Retinal arterioles are prone to damage in the form of stiffening and thickening. We therefore explored the occurrence of retinal arteriolosclerosis in patients with genetically verified FH, and in a control group. Associations between retinal arteriolosclerosis and current serum lipid values, sex, age, statin treatment, and years on lipid-lowering treatment were also investigated.

**Methods:**

Eligible patients were 40 to 70 years of age, normotensive, and non-smokers. Exclusion criteria were diabetes and vascular retinal disease. Control subjects had to have normal lipid levels and no history of cardiovascular disease. Lipid measurements were collected prior to the visit, and at the visit retinal fundus photographs of both eyes were taken. The fundus photographs were graded by two ophthalmologists according to the Scheie classification for retinal arteriolosclerosis.

**Results:**

Fifty patients with FH and 30 control subjects participated. Mean duration of lipid-lowering medication for the patient group was 17.0 years. The control group had higher total cholesterol, LDL-C and HDL-C, ApoB and ApoA1 levels than the patient group. There were no differences in the occurrence of retinal arteriolosclerosis between the two groups, and no associations were found between current lipid levels, age, sex, statin treatment, years on LLT and retinal arteriolosclerosis.

**Conclusion:**

Patients with FH did not have increased prevalence of retinal arteriolosclerosis compared to controls.

## Highlights

• 50 patients with FH and 30 controls investigated for retinal arteriolosclerosis.
• Retinal fundus photographs staged according to the Scheie classification.
• No increased retinal arteriolosclerosis in FH compared to controls.
• No associations with current lipid levels, age, sex and statin treatment.
• Retinal arterioles may not mirror macrovascular atherosclerotic changes.

## Graphical abstract

**Figure:**
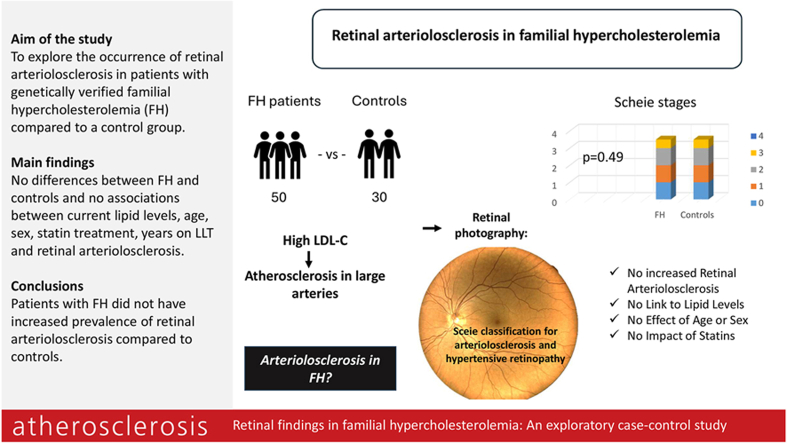

## 1 Introduction

Patients with familial hypercholesterolemia (FH) have elevated low-density lipoprotein-cholesterol (LDL-C) levels from birth, leading to increased risk of atherosclerosis and cardiovascular disease [1]. Atherosclerosis is a multifactorial disease mostly affecting middle to large arteries and is characterized by lipid accumulation, inflammation and matrix protein and calcium deposits in the artery wall, which can cause acute or chronic obstruction of the arterial lumen. Less is known about the effect of elevated LDL-C on arteriosclerosis in smaller arteries and arterioles [2]. Arteriolosclerosis is partly due to extravasation of plasma components across injured endothelial cells, leading to accumulation of proteins and thickening of the vascular wall and partly due to proliferation of smooth muscle cells and thickening of the vessel basement membrane [3,4]. Arteriolosclerosis is associated with hypertension (HT), diabetes mellitus (DM), and age [5].

FH-induced atherosclerosis primarily affects coronary arteries and mid-sized and large arteries, including the carotid arteries, abdominal aorta and lower limb arteries [6,7]*.* The influence of FH on smaller arterioles is less known, but it is reasonable to assume that if elevated LDL-C relates to arteriolosclerosis, it would manifest in patients with FH who have accumulated a significant LDL-C burden. In this regard, retinal arterioles can be directly and noninvasively examined by retinal fundus photography.

The primary aim of this study was to determine the occurrence of retinal arteriolosclerosis in patients with FH compared to controls. The secondary aims were to investigate the association between retinal arteriolosclerosis and current serum lipid values, sex, and age and to assess the association between retinal arteriolosclerosis, statin treatment and duration of lipid-lowering medication (LLM).

## 2 Methods

Inclusion criteria in the FH-group were: Genetically verified FH, age 40-70 years and initiation of lipid lowering therapy after 30 years of age, systolic blood pressure <160 mmHg, with no current use of antihypertensive medication. Exclusion criteria were: Body mass index (BMI) above 30 kg/m^2^, diabetes mellitus (HbA1c > 42 mmol/mol, active cancer disease or ongoing cancer treatment, acute or chronic infection or C-reactive protein (CRP) > 10 mg/L, undergone organ transplantation or on cytostatic or steroid treatment, vascular retinal disease, pregnancy or lactation and smoking. In the control group, inclusion and exclusion criteria were similar to those for the FH-group, except that LDL-C should be ≤ 4 mmol/L and triglycerides (TG) ≤ 1.7 mmol/L, without use of LLM in the last two months, and with no history of cardiovascular disease. Patients with FH were identified and recruited from an approved patient register at the Lipid Clinic, Oslo University Hospital, DYSLIPOUS (Reference 2013/16173). Controls were recruited via a website linked at the Oslo University Hospital intranet, with information about the study and a link to an online form. Patients and controls attended one study visit between October and December 2023 at Oslo University Hospital. A validated questionnaire, SmartDiet, assessing the heart-healthiness of the diet [8], and a customized questionnaire about medical history and lifestyle was completed. Examinations included non-mydriatic fundus photography of both eyes, measurements of blood pressure (BP), weight and height. A fasting blood draw was performed prior to the visit, either locally or at the Oslo University Hospital laboratory, with the following blood tests: TG, total cholesterol (TC), LDL-C, high-density lipoprotein-cholesterol (HDL-C), Apolipoprotein A1 (ApoA1), Apolipoprotein B (ApoB), Lipoprotein(a), glucose, aspartate aminotransferase (AST), alanine transaminase (ALT), CRP and HbA1c.

### 2.1 Retinal fundus photography

Non-mydriatic retinal fundus photographs of both eyes were obtained using a Clarus 500 camera (Carl Zeiss AG, Jena, Germany). One color photograph of each eye was taken and saved as a color photograph and a red-free copy. Two experienced ophthalmologists independently reviewed the fundus photographs and then jointly assigned grades according to the Scheie classification, which stages arteriolosclerosis and hypertensive retinopathy (Table 1) [10], resolving all discrepancies by consensus.

**Table 1:** The Scheie classification for arteriolosclerosis and hypertensive retinopathy.

	Arteriolosclerosis		Hypertensive Retinopathy
Stage 0	Normal	Grade 0	No visible abnormalities
Stage 1	Broadening of arteriolar light reflex	Grade 1	Diffuse arterial narrowing
Stage 2	Stage 1 + AV crossing changes	Grade 2	Stage 1 + focal arteriolar constriction
Stage 3	Copper wiring of arterioles	Grade 3	Stage 2 + retinal hemorrhage
Stage 4	Silver wiring of arterioles	Grade 4	Stage 4: Stage 3 + hard exudates* + retinal edema + optic disc swelling

AV = arteriovenous. *Hard exudates are lipid and proteinaceous material (e.g. fibrinogen and albumin) that leak from an impaired blood-retinal barrier [9].

### 2.2 Statistical analysis

The stage of retinal arteriolosclerosis was treated as an ordinal categorical variable with four levels. Group differences between participants with FH and controls were assessed using an ordinal logistic regression model (cumulative logit model). The model estimated the odds of being in a higher (more severe) Scheie stage in the FH group compared with the control group, assuming proportional odds across thresholds. The proportional odds assumption was evaluated and met. Results are presented as odds ratios (ORs) with 95% confidence intervals (CIs). Fisher's exact test was used to compare grades of hypertensive retinopathy. Normality of continuous variables was assessed using histograms, Q-Q plots, and comparison of medians and means. Between-group comparison was performed using the independent-samples Student's *t*-test for normally distributed continuous variables and the Wilcoxon rank-sum test for non-normally distributed variables. Pearson's correlation coefficient was used to assess associations between continuous variables. Ordinal logistic regression was used to estimate odds ratios (ORs) for associations between covariates and the degree of retinal arteriolosclerosis. Categorical variables are presented as frequencies and percentages, normally distributed continuous variables as means with standard deviations (SDs), and non-normally distributed variables as medians with 25th and 75th percentiles. Deviations from the total sample size are noted in the footnotes of the relevant tables. All statistical tests were two-sided, and a *P* value of <0.05 was considered statistically significant. Analyses were performed using Microsoft Excel (2016) and Stata/SE version 17.0.

## 3 Approvals

The study was conducted according to the principles of the Declaration of Helsinki version.

64, 2013 and was approved by the Regional Committees for Medical and Health Research.

Ethics (Reference 592700) and the Institutional Data Protection Officer at Oslo University Hospital (Reference 23/08292). All participants signed a digital informed consent form.

## 4 Results

### 4.1 Study population

In total, 247 subjects with FH were contacted by mail, 78 were interested in participating and 50 subjects met the inclusion criteria and were included in the study. As controls, 60 individuals completed the online interest form and 30 of these met the inclusion criteria and were included in the study. There were more women in both the FH group (58.0%) and the control group (76.7%). The FH group was on average 4.2 years older (57.4 versus 53.2 years), had a 1.5-kg/m^2^ higher BMI (24.7 versus 23.2 kg/m^2^) and had 6.2-mmHg higher systolic blood pressure (SBP) (122.5 versus 116.3 mmHg) than the control group (Table 2).

**Table 2:** Characteristics of study population.

	FH, n = 50	Control, n = 30	p-value*
**Women**	29 (58.0)	23 (76.7)	0.09
**Age,** years	57.4 ± 7.0	53.2 ± 8.2	**0.02**
**BMI,** kg/m^2^	24.7 ± 2.9	23.2 ± 2.9	**0.03**
**SBP,** mmHg	122.5 ± 14.0	116.3 ± 9.5	**0.03**
**Educational level**			
Middle school	1 (2.0)	0 (0.0)	
High school	3 (6.0)	1 (3.3)	
Vocational education	3 (6.0)	0 (0.0)	
College/university <4 years	17 (34.0)	9 (30.0)	0.63
College/university >4 years	26 (52.0)	20 (66.7)	
**Ethnicity**			
Caucasian	48 (96.0)	28 (93.3)	
Asian	1 (2.0)	2 (6.7)	
South American	1 (2.0)	0 (0.0)	0.72

All values are presented with mean ± SD or n (%). *p-values for differences between the FH-group and control group. Statistically significant values are presented in bold (p < 0.05). *Abbreviations: FH: familial hypercholesterolemia, BMI: body mass index, SBP: systolic blood pressure.*

Mean age at diagnosis of FH was 37.3 (SD 8.9) years. Mean age at start of LLM was 40.4 (SD 6.2) years and mean duration of LLM was 17.0 (SD 9.0) years. All patients except one currently received LLM, 74% were on high-intensity statins, 86% on ezetimibe and 28% on PCSK9-inhibitors. Mean duration of LLM was 17.0 (SD 9.0) years. Six patients had undergone percutaneous coronary intervention and five had suffered myocardial infarction (Table 3).

**Table 3:** Patient characteristics, diagnosis and lipid lowering medication.

	Total (n = 50)	Women (n = 29)	Men (n = 21)	p-value*
**Age,** years	57.4 ± 7.0	56.0 ± 7.1	59.2 ± 6.6	0.11
**Age of FH diagnosis**, years	37.3 ± 8.9	36.2 ± 9.3	38.7 ± 8.4	0.35
**Age of start LLM**, years	40.4 ± 6.2	40.4 ± 5.8	40.4 ± 6.9	0.99
**Duration of LLM treatment,** years	17.0 ± 9.0	15.6 ± 8.8	18.9 ± 9.0	0.21
**Number of LLM**				
No LLM	1 (2.0)	1 (3.4)	0 (0.0)	
Single LLM	4 (8.0)	0 (0.0)	4 (19.0)	
Double LLM	37 (74.0)	22 (75.9)	15 (71.4)	
Triple/quadruple LLM	8 (16.0)	6 (20.7)	2 (9.5)	**0.05**
**Statin treatment**	42 (84.0)	24 (82.8)	18 (85.7)	1.00
**Statin intensity**^a^				0.39
High intensity	31 (73.8)	16 (66.7)	15 (83.3)	
Moderate intensity	9 (21.4)	7 (29.2)	2 (11.1)	
Low intensity	2 (4.8)	1 (4.2)	1 (5.6)	
**Ezetimibe treatment**	43 (86.0)	27 (93.1)	16 (76.2)	0.12
**PCSK9 inhibitor treatment**	14 (28.0)	8 (27.6)	6 (28.6)	0.94
**Bile acid sequestrant**	4 (8.0)	3 (10.3)	1 (4.8)	0.63

**Other medication**				
Acetylsalicylic acid	12 (24.0)	5 (17.2)	7 (33.3)	0.19
Hormone replacement therapy		5 (17.2)		
**CVD**	12 (24.0)	6 (20.7)	6 (28.6)	0.52

All values are presented with mean ± SD or n (%). *p-values for differences between the men and women. Statistically significant values are in bold (p < 0.05). Time on LLM-treatment: n = 49.

a High intensity statin treatment: 40-80 mg Atorvastatin, 20-40 mg Rosuvastatin or 80 mg Simvastatin. Moderate intensity: 10-20 mg Atorvastatin, 5-10 mg Rosuvastatin, 20-40 mg Simvastatin, 40-80 mg Pravastatin or 40-80 mg Fluvastatin. Low intensity statin treatment: 5 mg Atorvastatin, 10 mg Simvastatin, 20 mg Pravastatin or 20-40 mg Fluvastatin [11]. *Abbreviations: FH: familial hypercholesterolemia, LLM: lipid lowering medication, PCSK9: proprotein convertase subtilisin/kexin type 9, CVD: cardiovascular disease.*

### 4.2 Blood lipids and other laboratory results

The control group had significantly higher TC, LDL-C, HDL-C, ApoB and ApoA1 levels than the patient group. HbA1c, AST and ALT were significantly higher in the patient group, but within normal ranges (Table 4).

**Table 4:** Plasma lipid levels and other blood sample parameters.

	FH n = 50	Control n = 30	p-value*
**TC,** mmol/L	4.5 ± 1.5	5.4 ± 0.8	**0.002**
**LDL-C,** mmol/L^a^	2.5 (2.0, 3.0)	3.6 (3.2, 4.1)	**<0.001**
**TG,** mmol/L^a^	0.866 (0.685, 1.075)	0.850 (0.710, 1.150)	0.84
**HDL-C,** mmol/L	1.5 ± 0.4	1.8 ± 0.4	**0.003**
**ApoA1,** g/L	1.44 ± 0.24	1.57 ± 0.22	**0.02**
**ApoB,** g/L^a^	0.8 (0.6, 0.9)	0.9 (0.8, 1.1)	**0.004**
**Lp(a),** mg/L^a^	106 (100, 209)	137 (100, 376)	0.428
**HbA1c,** mmol/mol	37.5 ± 4.2	35.7 ± 2.8	**0.04**
**ASAT,** U/l	24.3 ± 7.8	19.7 ± 7.4	**0.01**
**ALAT,** U/l	32.9 ± 13.1	26.1 ± 13.4	**0.03**
**Glucose,** mmol/L	5.0 ± 0.48	4.9 ± 0.40	0.59
**CRP,** mg/L^a^	0.6 (0.3, 0.8)	0.65 (0.3, 1.3)	0.31

All values are presented with mean ± SD or n (%) if not otherwise stated. *p-values for differences between the FH-group and control group. Statistically significant values are in bold (p < 0.05).

a Values are presented with median, 25th and 75th percentiles in parenthesis. TG: n = 48 in the FH group and n = 29 in the control group. ApoB, HbA1c, ASAT and ALAT: n = 49 in FH group. ASAT and glucose: n = 29 in control group. Glucose: n = 44 in FH group. Lp(a) n = 35 in FH group and n = 16 in control group. *Abbreviations: TC: total cholesterol, LDL-C: low-density lipoprotein cholesterol, TG: triglyceride, HDL-C: high-density lipoprotein cholesterol, ApoA1: apolipoprotein A1, ApoB: Apolipoprotein B, Lp(a): lipoprotein(a), HbA1c: hemoglobin A1c, ASAT: Aspartate aminotransferase, ALAT: Alanine aminotransferase, CRP: C-reactive protein.*

### 4.3 Retinal arteriolosclerosis

The distribution of Scheie stages was similar between the FH and control groups (Table 5 and Supplementary Fig. 1). In ordinal regression analysis, the odds of having a higher degree of arteriolosclerosis were similar in patients with FH and controls (OR, 0.75; 95% CI, 0.33–1.71; p = 0.49). This finding remained unchanged after adjusting for sex, age, and BMI (adjusted OR 0.76; p = 0.53). No association was observed between the grade of retinal arteriolosclerosis and systolic blood pressure in the total population (OR 0.99; CI 0.96-1.03; p = 0.71) or among patients with FH (OR 1.00; 95% CI 0.96-1.04; p = 0.91). Similarly, no associations were found between retinal arteriolosclerosis grade and current LDL-C levels in the total population or between years on LLM, statin treatment or CVD within the FH-group (Supplementary Tables 1 and 2)

**Table 5:** Retinal arteriolosclerosis stages in the total study population, familial hypercholesterolemia group, and control group (Scheie classification).

	Total, n (%)	FH, n (%)	Control, n (%)
**Stage 0**	20 (25.0)	13 (26.0)	7 (23.3)
**Stage 1**	23 (28.8)	15 (30.0)	8 (26.7)
**Stage 2**	25 (31.3)	16 (32.0)	9 (30.0)
**Stage 3**	12 (15.0)	6 (12.0)	6 (20.0)
**Stage 4**	0 (0.0)	0 (0.0)	0 (0.0)

### 4.4 Hypertensive retinopathy

There was no significant difference in the distribution of the Scheie grades of hypertensive retinopathy between the FH-group and the control group (Fischer's exact test, p = 0.39) (Supplemental Fig. 2).

## 5 Discussion

Our study did not show increased prevalence of retinal arteriolosclerosis in patients with FH compared with controls. Neither did we find an association between current lipid profile and degree of retinal arteriolosclerosis in the entire group. Furthermore, there were no associations between statin treatment, duration of LLM or prevalence of CVD and arteriolosclerosis in the FH group.

To ensure a difference in lifelong LDL-C load between the FH-group and the control group, FH-patients should not have initiated LLT before 30 years of age. Mean age at start of LLT in the FH group was 40 years and mean duration of LLT was 17 years. Assuming at least 50% mean reduction in LDL-C levels when treated, LDL-C burden among FH patients could be conservatively approximated as: 0-20 years of age: mean LDL-C 4.0 mmol/L = 80.0 mmol-years; 20-40 years of age: mean LDL-C 5.0 mmol/L = 100.0 mmol-years; from 40 to 57 years of age: treated LDL-C 2.5 mmol/L = 42.5 mmol-years, summing up to 222.5 mmol-years [12]. For the control group, based on estimated mean population levels of LDL-C as shown in the Lifelines studies [13,14], a similar approximation would be: 0-20 years of age 2.5 mmol/L x 20 = 50.0 mmol-years; 20-30 years of age 3.0 mmol/L x 10 = 30.0 mmol-years and 30-53 years of age: 3.6 mmol/L x 23 = 82.8 mmol-years, summing up to 162.8 mmol-years. Consequently, there was a higher lifelong LDL-C load among FH-patients than in controls, suggesting no clear association between LDL-C levels and arteriolosclerosis.

Evidence regarding retinal abnormalities in patients with FH is scarce. Contrary to our findings, a small study found more arterio(lo)sclerosis in patients with severe FH compared with controls according to the Scheie grading for hypertensive retinopathy, independent of LDL-C levels [15]. Another small cross-sectional case-control study reported a correlation between retinal arteriolosclerosis and TC, LDL-C, TG, and ApoB among middle-aged (non-FH) men, compared to matched, apparently healthy controls without retinal changes [16].

The Scheie classification is a relatively coarse method for assessing the retinal vasculature and may be insufficiently sensitive to detect subtle microvascular alterations. In contrast, optical coherence tomography angiography (OCTA) provides a more detailed evaluation of the retinal microcirculation. Using OCTA, reduced retinal parafoveal vascular density has been shown to be associated with subclinical coronary atherosclerosis, supporting the concept that retinal microvascular changes may reflect early systemic vascular disease [17]. Moreover, altered microvascularization has been demonstrated in patients with FH. [18]. In a small study in patients with severe FH undergoing lipoprotein apheresis, optic coherence tomography detected significantly thinner superior and inferior retinal nerve fibre layer (RNFL), resulting in reduced global RNFL thickness, and a significantly larger foveal avascular zone than controls, reflecting early signs of retinal vessel damage [19]. Probably, many of these patients on lipid apheresis had substantially elevated cholesterol levels contributing to the findings. Hyperlipidemia is also a recognized systemic vascular risk factor for retinal vein occlusive disease, further supporting the biological plausibility of dyslipidemia-related retinal vascular changes [20,21].

Some large cohort studies have explored associations between dyslipidemia and retinal abnormalities and concluded that elevated LDL-C or dyslipidemia do not appear to impact retinal abnormalities [[22], [23], [24], [25], [26]], supporting the results in our study. The lack of an association between elevated LDL-C and arteriolosclerosis may be explained by anatomical and physiological differences between arterioles and larger arteries. Arterioles have a thinner intima and a higher proportion of smooth muscle cells in the media, whereas larger arteries have thicker walls with more elastic fibers. In large arteries, lipid particles penetrate the endothelium, triggering inflammation and atheromatous plaque formation within the tunica intima and media. Higher hydrostatic pressure in proximal arteries likely further promotes lipid accumulation within the vessel wall. In contrast, arterioles lack the wall layers in which lipid particles are typically retained, and the lower hydrostatic pressure may inhibit atherosclerosis development [27,28]. Arteriolosclerosis, which is characterized by stiffening and hardening of the vessel wall, is instead driven primarily by smooth muscle cell proliferation and serum protein deposition in the tunica media, with lipids and inflammation playing a limited role [3].The fact that there is no increased risk of ischemic cerebral events in patients with FH may also be because these events are caused by changes in smaller cerebral arteries and arterioles, more related to hypertension and age and not affected by elevated LDL-C and inflammation to the same extent as larger arteries [29].

## 6 Strengths and limitations

The small sample size and limited statistical power is an obvious limitation. Another weakness is the subjective nature of retinal arteriolosclerosis staging, although assessments were performed by two experienced ophthalmologists. Moreover, although staging of retinal arteriolosclerosis according to the Scheie classification is validated, it is more commonly applied in the context of hypertensive retinopathy. Despite these limitations, the findings of this study are relevant and warrant comparison and replication in larger future studies with more refined methods for assessing retinal vasculature.

## 7 Conclusion

No increased prevalence of retinal arteriolosclerosis, as assessed by the Scheie classification, was observed in patients with FH compared with controls. However, because the Scheie classification may lack sensitivity for detecting subtle microvascular changes, these findings should be interpreted with caution.

## Conflicts of interest

GL received consultant honoraria from Ultragenyx. KR received payments or honoraria for lectures from: Amgen, Amarin, MSD, Novartis, Norwegian Directorate of Health, The Norwegian Medical Products Agency and Sanofi. ØKJ received consultant or speaker honoraria from AbbVie, Bayer, Chiesi Farmaceutici and Horus Pharma, research support from Haag-Streit, consultant honoraria and funding from Roche, and consultant, speaker honoraria and royalties from SJJ Solutions. KBH has received honoraria or consulting fees for professional input and/or delivered lectures from European Atherosclerosis Society, Sanofi, Ultragenyx, Amgen and Menarini. SK and MGE have nothing to declare. The study was funded by the Institute of Basic Medical Sciences, University of Oslo.
