# Supplementary material for: Retinal findings in familial hypercholesterolemia: An exploratory case-control study
Source: Atheroscler Plus. 2026 Jun 4;65:100571. doi: 10.1016/j.athplu.2026.100571 (PMC13273735; doi:10.1016/j.athplu.2026.100571)
Supplement: Multimedia component 1 [file mmc1.docx]

**Supplementary material**

**Supplementary table 1.** Odds ratio (OR) and 95% confidence intervals for the association between grade of retinal arteriolosclerosis and different factors.

|  | OR | 95% CI | p-value* |
| --- | --- | --- | --- |
| **Entire population** | | | |
| FH patient | 0.75 | 0.33-1.71 | 0.49 |
| Women | 1.49 | 0.65-3.42 | 0.35 |
| Age | 1.00 | 0.95-1.05 | 0.96 |
| Lp(a)^§^ | 1.00 | 1.00-1.00 | 0.72 |
| LDLc | 1.08 | 0.81-1.43 | 0.61 |
| TC | 1.10 | 0.82-1.46 | 0.52 |
| TG | 1.05 | 0.35-3.21 | 0.93 |
| HDLc | 1.09 | 0.41-2.88 | 0.87 |
| HbA1c | 1.04 | 0.93-1.16 | 0.48 |
| **FH population** | | | |
| Women | 2.75 | 0.81-6.66 | 0.08 |
| Age | 0.99 | 0.92-1.06 | 0.79 |
| Lp(a) ^§^ | 1.00 | 1.00-1.00 | 0.90 |
| LDLc | 1.04 | 0.75-1.45 | 0.81 |
| Years on LLM | 0.97 | 0.92-1.03 | 0.31 |
| Statins | 2.80 | 0.66-11.8 | 0.16 |
| Average cholesterol burden | 1.04 | 0.72-1.51 | 0.83 |

^§^n=51 in the entire population and n=35 in the FH population..

*Abbreviations: FH: familial hypercholesterolemia, HbA1c: haemoglobin A1c, HDL-C: high-density lipoprotein*

*cholesterol, LDL-C: low-density lipoprotein cholesterol, LLM: lipid-lowering medication, Lp(a): lipoprotein(a),*

*TC: total cholesterol, TG: triglyceride.*

| **Supplementary table 2.** Odds ratios and 95% confidence intervals for the association between different  lifestyle factors and grade of retinal arteriolosclerosis.   \|  \| Entire population \| \| \| FH patients \| \| \| \| --- \| --- \| --- \| --- \| --- \| --- \| --- \| \|  \| OR \| 95% CI \| p-value \| OR \| 95% CI \| p-value \| \| **HT retinopathy** \| 2.38 \| 1.08-5.26 \| **0.03** \| 2.69 \| 1.03-7.00 \| **0.04** \| \| **SBP** \| 0.99 \| 0.96-1.03 \| 0.71 \| 1.00 \| 0.96-1.04 \| 0.91 \| \| **SmartDiet score** \| 1.13 \| 1.00-1.28 \| ***0.06*** \| 1.07 \| 0.92-1.24 \| 0.40 \| \| **>4 years of higher education** \| 0.70 \| 0.31-1.60 \| 0.39 \| 0.57 \| 0.21-1.60 \| 0.29 \| \| **Years of smoking** \| 1.04 \| 0.93-1.17 \| 0.46 \| 1.11 \| 0.96-1.30 \| 0.16 \| \| **BMI** \| 0.98 \| 0.85-1.14 \| 0.83 \| 1.08 \| 0.90-1.30 \| 0.41 \|   Results are after adjusting for sex, age, BMI or FH diagnosis. Statistically significant values are in bold (p<0.05).  P-values between 0.05 and 0.1 are in bold italic. Years of smoking: n=31 in entire population and n=21 within FH-group.  *Abbreviations: BMI: body mass index. FH: familial hypercholesterolemia, HT: hypertension, SBP:*  *systolic blood pressure.* | | | | |  |
| --- | --- | --- | --- | --- | --- | --- | --- | --- | --- | --- | --- | --- | --- | --- | --- | --- | --- | --- | --- | --- | --- | --- | --- | --- | --- | --- | --- | --- | --- | --- | --- | --- | --- | --- | --- | --- | --- | --- | --- | --- | --- | --- | --- | --- | --- | --- | --- | --- | --- | --- | --- | --- | --- | --- | --- | --- | --- | --- | --- | --- | --- |
| **Supplementary figure 1**  Retinal arteriolosclerosis stages in the familial hypercholesterolemia (FH) group and control group (Scheie classification)  **Supplemental Figure 2**  Distribution of the different grades of hypertensive retinopathy in percentage in the familial hypercholesterolemia (FH) group and the control group. Grading is according to the Scheie classification for hypertensive retinopathy. P-value for difference between groups, Fisher exact test = 0.39. |  |  |  |  |  |
